# Treatment as Required versus Regular Monthly Treatment in the Management of Neovascular Age-Related Macular Degeneration: A Systematic Review and Meta-Analysis

**DOI:** 10.1371/journal.pone.0137866

**Published:** 2015-09-14

**Authors:** Christine M. Schmucker, Gerta Rücker, Harriet Sommer, Gianni Virgili, Yoon K. Loke, Patrick Oeller, Hansjuergen Agostini, Christoph Ehlken

**Affiliations:** 1 Cochrane Germany, Medical Center – University of Freiburg, Berliner Allee 29, 79110 Freiburg, Germany; 2 Institute for Medical Biometry and Statistics, Medical Center – University of Freiburg, Stefan-Meier-Str. 26, 79104 Freiburg, Germany; 3 Department of Translational Surgery and Medicine, University of Florence, Florence, Italy; 4 Norwich Medical School, University of East Anglia, Norwich, United Kingdom; 5 University Eye Center Freiburg, Killianstr. 5, 79106 Freiburg, Germany; Copenhagen University Hospital Roskilde and the University of Copenhagen, DENMARK

## Abstract

**Background:**

To investigate whether treatment as required ‘pro re nata’ (PRN) versus regular monthly treatment regimens lead to differences in outcomes in neovascular age-related macular degeneration (nAMD). Regular monthly administration of vascular endothelial growth factor (VEGF) inhibitors is an established gold standard treatment, but this approach is costly. Replacement of monthly by PRN treatment can only be justified if there is no difference in patient relevant outcomes.

**Methods:**

Systematic review and meta-analysis. The intervention was PRN treatment and the comparator was monthly treatment with VEGF-inhibitors. Four bibliographic databases were searched for randomised controlled trials comparing both treatment regimens directly (head-to-head studies). The last literature search was conducted in December 2014. Risk of bias assessment was performed after the Cochrane Handbook for Systematic Reviews of Interventions.

**Findings:**

We included 3 head-to-head studies (6 reports) involving more than 2000 patients. After 2 years, the weighted mean difference in best corrected visual acuity (BCVA) was 1.9 (95% CI 0.5 to 3.3) ETDRS letters in favour of monthly treatment. Systemic adverse events were higher in PRN treated patients, but these differences were not statistically significant. After 2 years, the total number of intravitreal injections required by the patients in the PRN arms were 8.4 (95% CI 7.9 to 8.9) fewer than those having monthly treatment. The studies were considered to have a moderate risk of bias.

**Conclusions:**

PRN treatment resulted in minor but statistically significant decrease in mean BCVA which may not be clinically meaningful. There is a small increase in risk of systemic adverse events for PRN treated patients. Overall, the results indicate that an individualized treatment approach with anti-VEGF using visual acuity and OCT-guided re-treatment criteria may be appropriate for most patients with nAMD.

## Introduction

Age-related macular degeneration (AMD) is a progressive and chronic disease of the retina that affects older adults. The loss of visual perception occurs primarily in the late stages of the disease due to neovascularisation, geographic atrophy, or a combination of the two processes. Intravitreal treatment with ranibizumab (Lucentis; Genentech, Inc., South San Francisco, CA), an antibody to vascular endothelial growth factor (VEGF), was shown to be more effective in neovascular AMD (nAMD) compared with photodynamic therapy[1, 2] or no treatment.[3] Intravitreal VEGF inhibition with either ranibizumab, bevacizumab, or aflibercept was thus established as the standard-of-care treatment option for the management of nAMD.

The pivotal studies, Anti-VEGF Antibody for the Treatment of Predominantly Classic Choroidal Neovascularization (CNV) in nAMD (ANCHOR)[1, 2] and Minimally Classic/Occult Trial of the Anti-VEGF Antibody Ranibizumab in the Treatment of nAMD (MARINA)[3], were the first randomised phase 3 clinical trials to demonstrate that monthly administration of 0.3 mg and 0.5 mg ranibizumab not only prevented vision loss associated with nAMD, but also improved mean visual acuity between 7.2 and 10.7 letters according to the Early Treatment Diabetic Retinopathy Study (ETDRS) over 2 years. The Phase IIIb, Multicenter, Randomized, Double-Masked, Sham Injection-Controlled Study of the Efficacy and Safety of Ranibizumab in Subjects with Subfoveal CNV with or without Classic CNV Secondary to nAMD (PIER) study[4] demonstrated that visual acuity outcomes were markedly better in patients receiving ranibizumab on a monthly basis compared to those assigned to 3 monthly loading doses, followed by prescheduled quarterly injections. Hence, the prescribing information for ranibizumab in Europe recommends monthly injections for optimal visual acuity outcomes.

However, frequent injections and assessments place a significant burden on patients and caregivers, and carry the risk of rare but serious ocular adverse events, e.g. endophthalmitis, associated with intravitreal injections.[5] Therefore, many retina specialists in clinical practice advocate individualized treatment regimens in an effort to reduce patient and caregiver burden and costs. In individualized variable dosing regimens such as pro re nata (PRN; as needed) the drug is—based predominantly on optical coherence tomography (OCT) and visual acuity findings—injected less frequently as long as there is no recurrence of neovascular manifestations. It is important to define the clinical value of these new approaches in managing nAMD, and as such, we conducted a systematic review of head-to-head trials comparing efficacy and safety outcomes between monthly and PRN anti-VEGF dosing regimens.

## Material and Methods

We included head-to-head randomised controlled trials (RCTs) comparing monthly (continuous) with PRN (discontinuous) anti-VEGF treatment. Eligible participants were individuals with nAMD of any phenotype, irrespective of age, sex, comorbidity, and diseases progression. A review protocol can be accessed from the corresponding author (CMS).

Primary outcome domains included changes in best corrected visual acuity (BCVA) from baseline and number of anti-VEGF injections at 2 years. Additionally, we investigated change in total lesion thickness at the fovea. Our safety outcomes included all-cause deaths and all serious systemic adverse events (i.e., the sum of individuals affected by 1 or more serious systemic adverse events such as occurrences that result in death, are life-threatening, require hospital admission or prolongation of hospital stay, cause persistent or significant disability/incapacity, or are medically important events or reactions). Additionally, we investigated arteriothrombotic events, defined as any patient who has experienced myocardial infarction, non-haemorrhaging stroke, angina, ischaemic heart disease, thrombosis, or death from cardiovascular disease. We collected outcomes at the maximum follow-up times reported up to 2 years.

Published studies were identified from searches of electronic databases. We searched Medline (OvidSP), Science Citation Index, PubMed-subset “supplied by publisher”, and the Cochrane Central Register of Controlled Trials from inception until September 2013. An update search was performed in December 2014. The search strategy was based on combinations of medical subject headings (MeSH) and keywords and was not restricted to specific languages. The search strategy used in Medline (OvidSP) is presented in S1 Search Strategy. Search strategies for other databases were modified to meet the requirements of each database. Although not the focus of this review, the literature search also included terms associated with diabetic macular edema (DME). These studies will contribute to a network meta-analysis (as a second phase of this project), to address the issue of the relative effectiveness and safety across a network of RCTs testing different anti-VEGF agents and treatment regimen in different indications. The searches were supplemented by handsearching the bibliographies of included studies and relevant systematic reviews. Potential ongoing studies were identified in clinicaltrials.gov.

The reviewers (CMS, CE, PO) independently screened the titles and abstracts of all reports identified by electronic searches. We obtained full-text copies of all potentially relevant articles and 2 reviewers independently assessed them for inclusion. These reviewers independently assessed the risk of bias of included full-text studies following the criteria outlined in Chapter 8 of the Cochrane Handbook for Systematic Reviews of Interventions[6], which addressed the following key domains: randomisation sequence generation, allocation concealment, masking (blinding) of participants, trial personal, and outcome assessors in terms of treatment regimen, incomplete outcome data, selective outcome reporting (e.g., absence of data for outcome measurements), and other sources of bias (e.g., bias due to problems not covered elsewhere). We evaluated additional risk of bias items specific to adverse events using the following items: 1) adverse event definition (if the definition of adverse events was pre-specified and collected based on standard criteria or classification systems (e.g., MedDRA SOC) and 2) method of adverse events assessment (if the researchers actively monitored for adverse events or simply provided spontaneous reporting of adverse events that arose during the study). Individual participants served as the unit of analysis.

We used weighted mean differences based on a fixed-effect model to estimate relative efficacy (visual acuity, morphological outcomes) and treatment frequency of the 2 treatment regimen. Due to the scarcity of studies in this field, we anticipated that the low number of RCTs in a pairwise comparison of the treatment regimens would prevent the formal assessment of statistical heterogeneity. Nevertheless, we calculated the Chi² and I² statistics.[7] Similarly, the paucity of RCTs in a pairwise comparison would also prevent the formal assessment of publication bias.

To evaluate safety outcomes, we reasoned that there may be true differences across the population of potential studies as they may have enrolled participants at different risk levels for adverse events. For instance, some studies might have included participants at high risk for arterial thromboembolic events, while others may have excluded such participants. For this reason, we used a Mantel-Haenszel-random-effects model to calculate the risk ratio (RR) for meta-analyses for safety data, which provides a robust estimate when pooling sparse data.

We carried out the analyses, as far as possible, on an intention-to-treat basis using the Review Manager Software.

## Results

### Study characteristics

**Fig 1:**
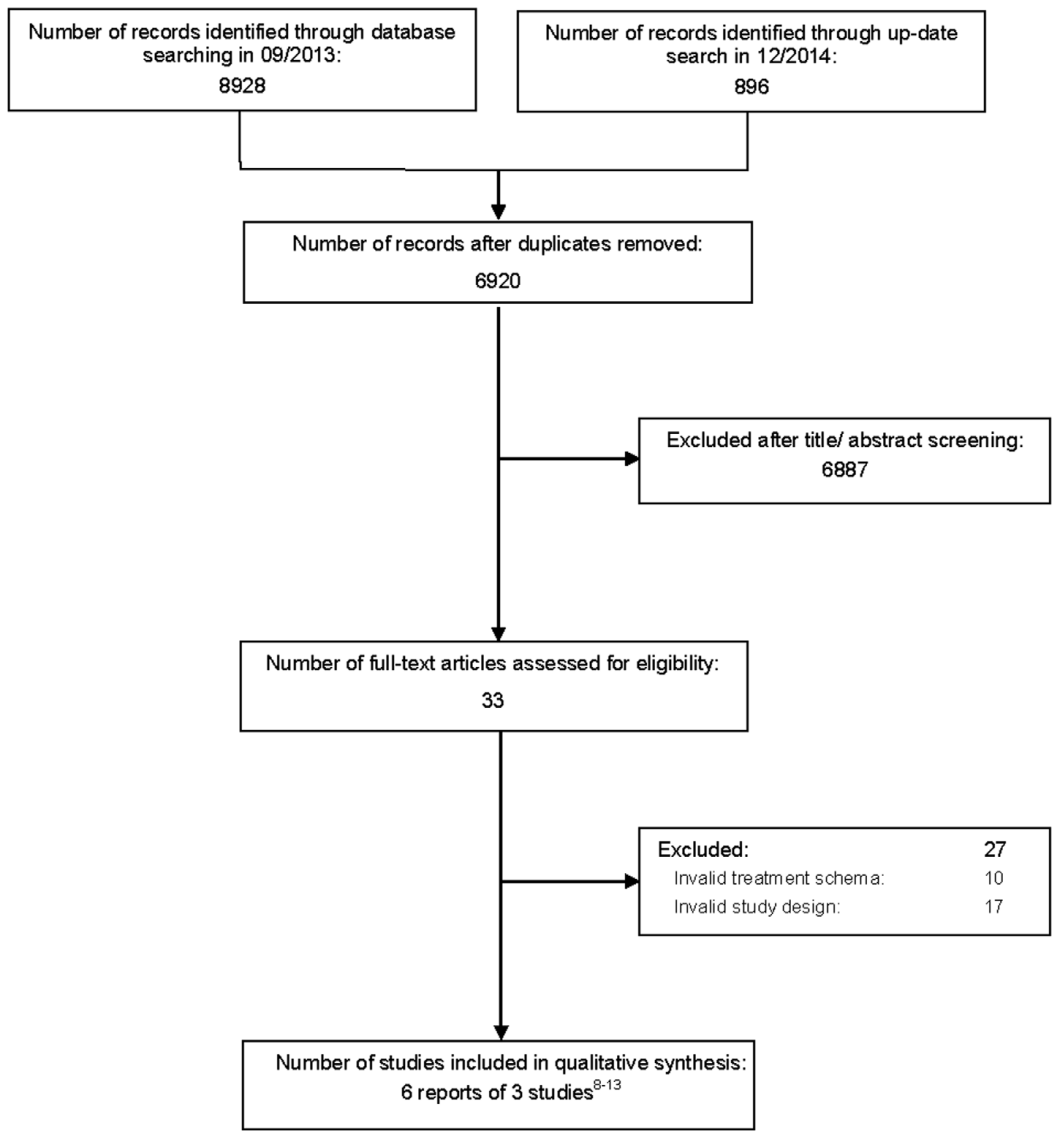
Flow diagram outlining the screening and selection process of articles.

We identified 9824 titles and abstracts; for 33 of these, the full text was evaluated. Fig 1 outlines the screening and selection process of the articles. Studies excluded after full-text screening are listed in S1 Excluded Studies.

Three studies (6 reports, including 1844 patients) were randomised head-to-head studies comparing PRN versus monthly treatment regimens over 2 years (Table 1).[8–13] Two of the studies were conducted in the USA (CATT,[8, 9] HARBOR [The Phase III, Double-Masked, Multicenter, Randomized Active Treatment-controlled Study of the Efficacy and Safety of 0.5 mg and 2.0 mg Ranibizumab Administered Monthly or on an As-Needed Basis in Patients with Subfoveal nAMD])[12, 13] and one in Europe (IVAN).[10, 11] One of these trials was sponsored by the pharmaceutical industry.[12, 13]

**Table 1:** Summary of the characteristics of the included head-to-head studies.

Study	Country	Enrollment	Intervention	Maximum follow-up (months)	No of patients PRN treated	No of patients monthly treated	PRN treatment schedule	OCT-Typ
CATT 2011/12	USA	02/08-12/09	RAB 0.5 mg or BEV 1.25 mg	12/24	598/515*	587/263*	One lading dose. Thereafter, patients were evaluated for treatment every 4 weeks and were treated when fluid was present on OCT or when new or persistent hemorrhage, decreased visual acuity relative to the previous visit, or dye leakage on FAG was present. Patients received 1 injection each time they met the retreatment criteria.	TD-OCT or SD-OCT**
HARBOR 2013/14	USA	03/08-10/10	RAB 0.5 mg	12/24	275	274	Three consecutive monthly loading doses. Thereafter, patients were evaluated every 4 weeks and treated when BCVA decreased by ≥5 letters compared to the previous visit or if there was any evidence of disease activity on SD-OCT. Patients received 1 injection each time they met the retreatment criteria.	SD-OCT
IVAN 2012/13	UK	03/08-10/10	RAB 0.5 mg or BEV 1.25 mg	12/24	302/258	308/259	Three consecutive monthly loading doses. Thereafter, patients were evaluated for treatment every 4 weeks and were treated when any subretinal fluid, intraretinal fluid, or fresh blood was visible. Also, if there was uncertainty about these criteria and visual acuity dropped by ≥ 10 letters. In the absence of fluid or visual acuity deterioration, fluorescein leakage >25% of the lesion circumference or expansion of CNV was required to initiate retreatment. Patients received 3 monthly injecions each time they met the retreatment criteria.	TD-OCT

BCVA = best corrected visual acuity; CATT = comparison of age related macular degeneration treatment trials; HARBOR = the phase III, double-masked, multicenter, randomized active treatment-controlled study of the efficacy and safety of 0,5 mg and 2,0 mg ranibizumab administered monthly or on an as-needed basis in patients with subfoveal neovascular age-related macular degeneration; IVAN = inhibit VEGF in age related choroidal neovascularisation; OCT = optical coherence tomography; PRN = pro re nata; SD = spectral domain, TD = time domain.

*In CATT, patients were assigned equally to 1 of 4 treatment groups defined by drug (ranibizumab or bevacizumab) and by dosing regimen at enrollment. At 1 year, patients initially assigned to monthly treatment retained their drug assignment but were reassigned randomly, with equal probability, to either monthly or PRN treatment. Patients initially assigned to PRN had no change in assignment.

**22.6% of the scans in the 2^nd^ year with SD-OCT.

Study participants had no previous treatment for nAMD. The included patients showed a variable baseline prevalence of cardiovascular risk factors such as a history of myocardial infarctions, strokes or transient ischemic attacks which ranged between 2.2% and 12%. In CATT and IVAN, the efficacies of ranibizumab 0.5 mg and bevacizumab 1.25 mg were compared using both PRN and monthly treatment regimens. In HARBOR ranibizumab 0.5 mg was the treatment of choice. Because different meta-analyses have shown that there is no difference between ranibizumab and bevacizumab in efficacy[10, 11] and safety[14] outcomes, we pooled ranibizumab and bevacizumab data together. Patients of the HARBOR and IVAN trials received 3 consecutive monthly loading doses before the actual PRN treatment started. CATT featured a single loading dose.

### Risk of bias

**Fig 2:**
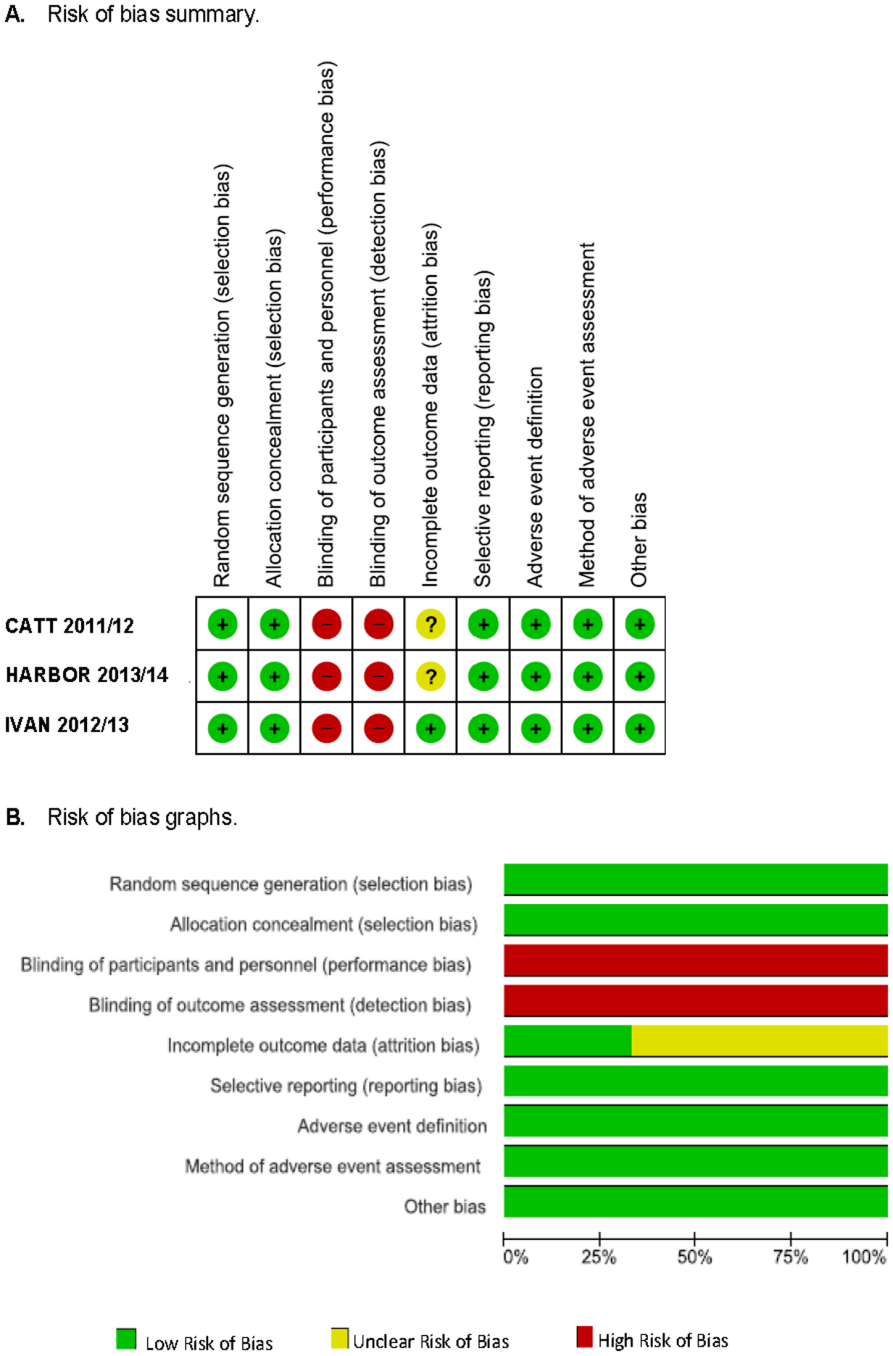
Risk of bias assessment.

The risk of bias summary for each study is presented in Fig 2A. The studies generally reported details about random sequence generation and allocation concealment. However, all of the treated patients and examiners were aware of their treatment regimen assignment. Two studies[8, 9, 12, 13] showed an unclear risk of bias concerning missing outcome data (due to drop-outs[8, 9, 12, 13] and/or re-randomisation after 1 year[8, 9]). All studies reported our primary outcomes and appeared to have implemented an appropriate, pre-specified definition of adverse events and actively monitored them. Fig 2B shows the risk of bias graph over all studies.

### Change in visual acuity

**Fig 3:**
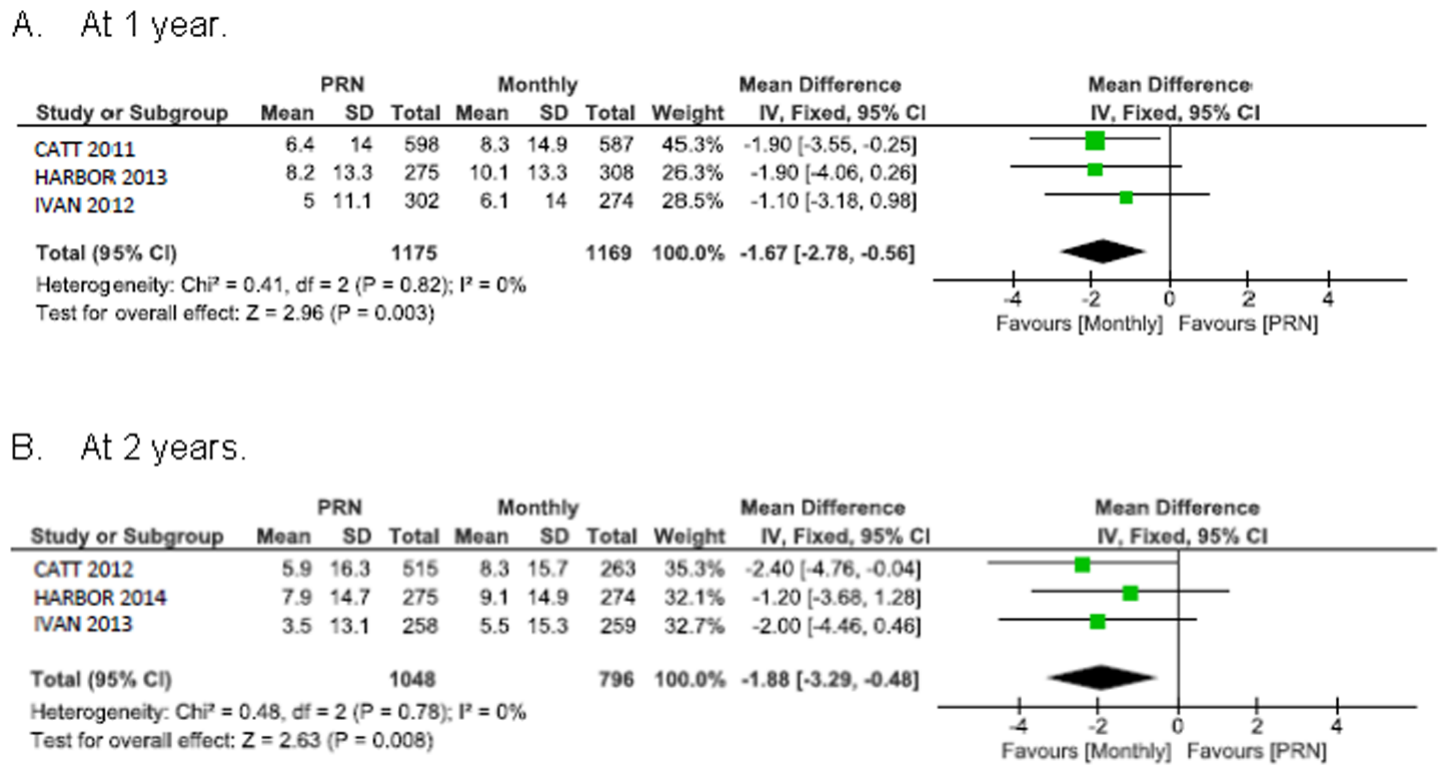
Mean change in best corrected visual acuity (BCVA) from baseline in ETDRS letters.

Visual acuity improved less with PRN treatment than with monthly treatment: 1.7 ETDRS letters in favour of monthly treatment, 95% CI 0.6 to 2.8 at 1 year; 1.9 ETDRS letters in favour of monthly treatment, 95% CI 0.5 to 3.3 at 2 years (Fig 3). These intervals lie within the CATT, HARBOR and IVAN non-inferiority range (margins of 3.5, 4.0, and 5.0 letters, respectively).

### Central retinal thickness

**Fig 4:**
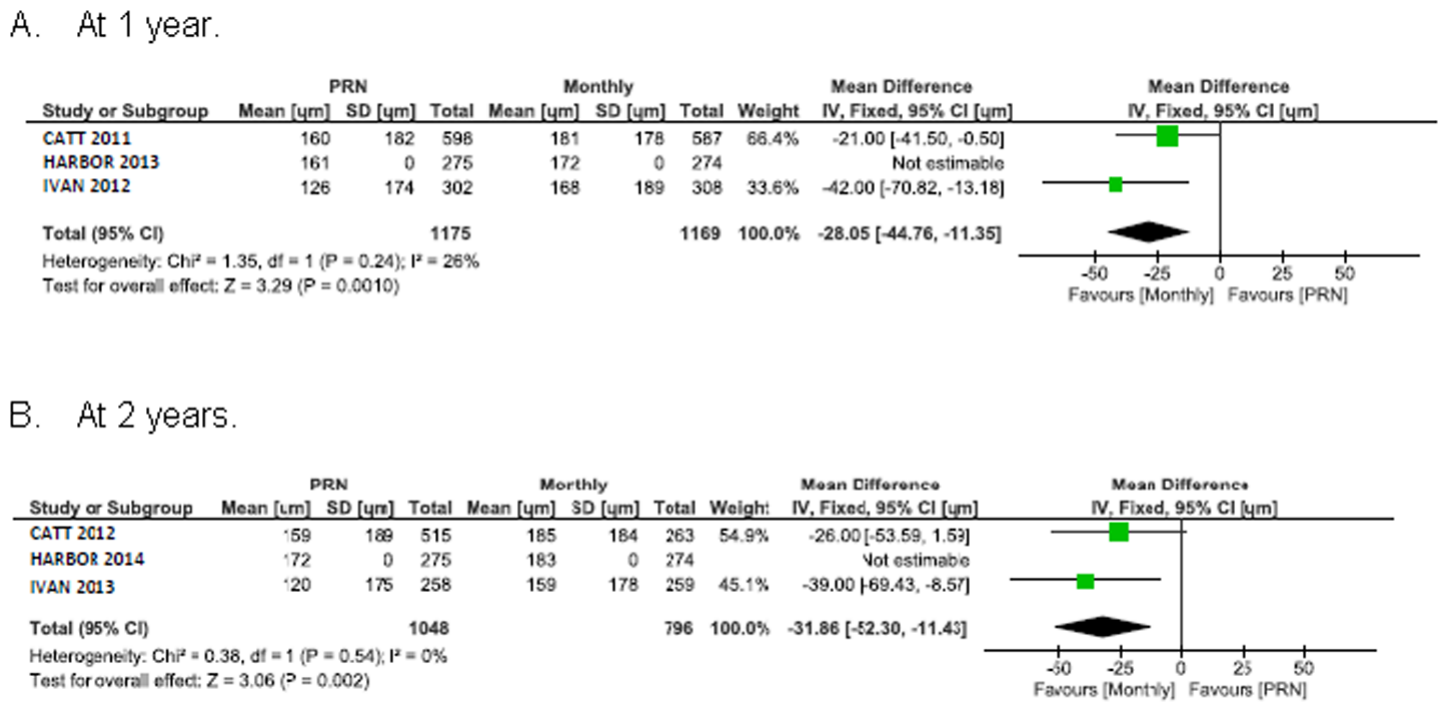
Mean decrease in central foveal thickness (CFT) from baseline in μm.

Pooled analysis of total central retinal thickness shows a significant difference of 28 μm (95% CI 11 to 45) favouring monthly treatment at 1 year (Fig 4). This difference was maintained at 2 years (32 μm [95% CI 11 to 52]).

### Treatment frequency

**Fig 5:**
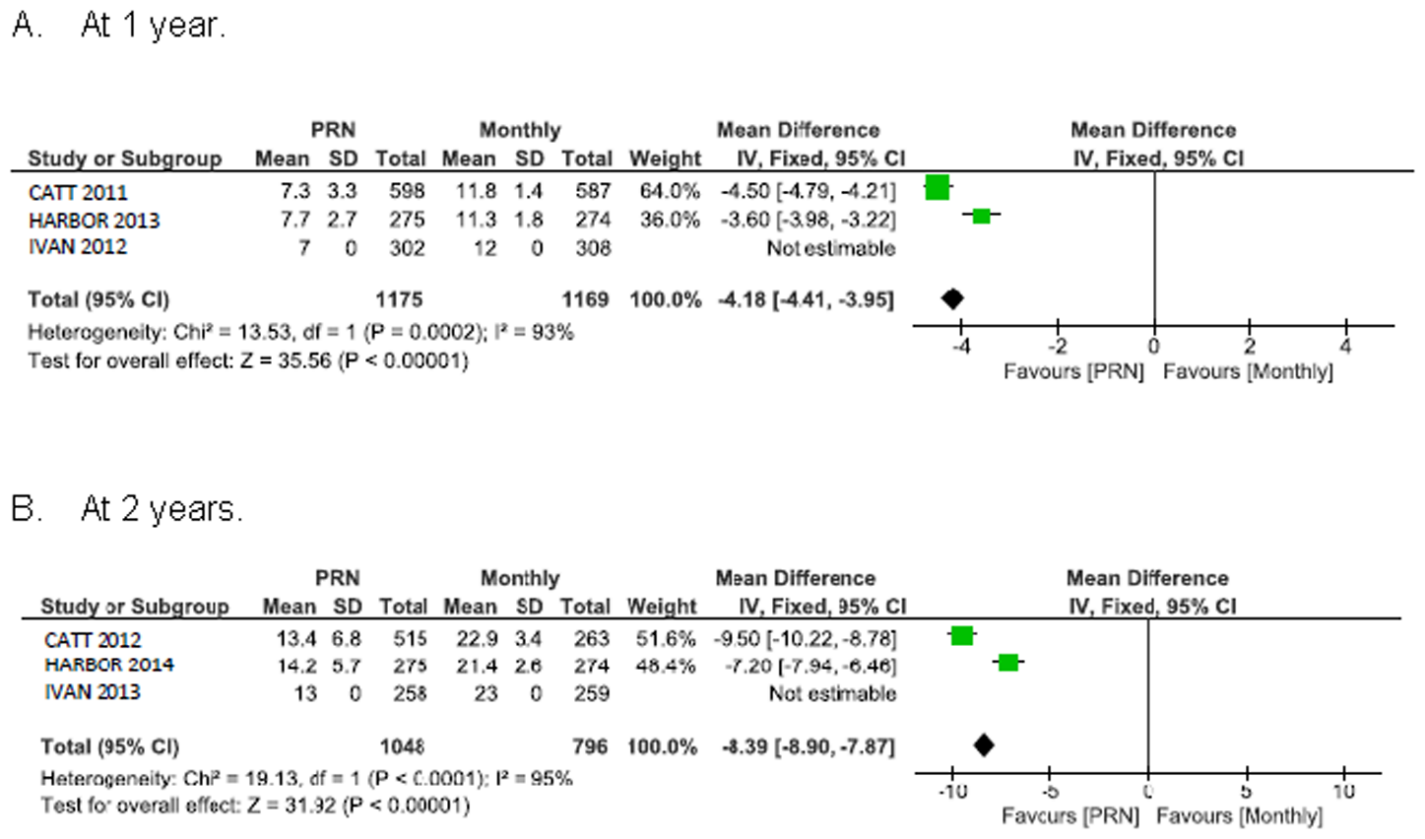
Mean difference in the number of anti-VEGF injections.

After 2 years, patients treated according to PRN regimen required 8.4 (95% CI 7.9 to 8.9) intravitreal injections less than monthly treated patients (Fig 5).

### Systemic safety outcomes

**Fig 6:**
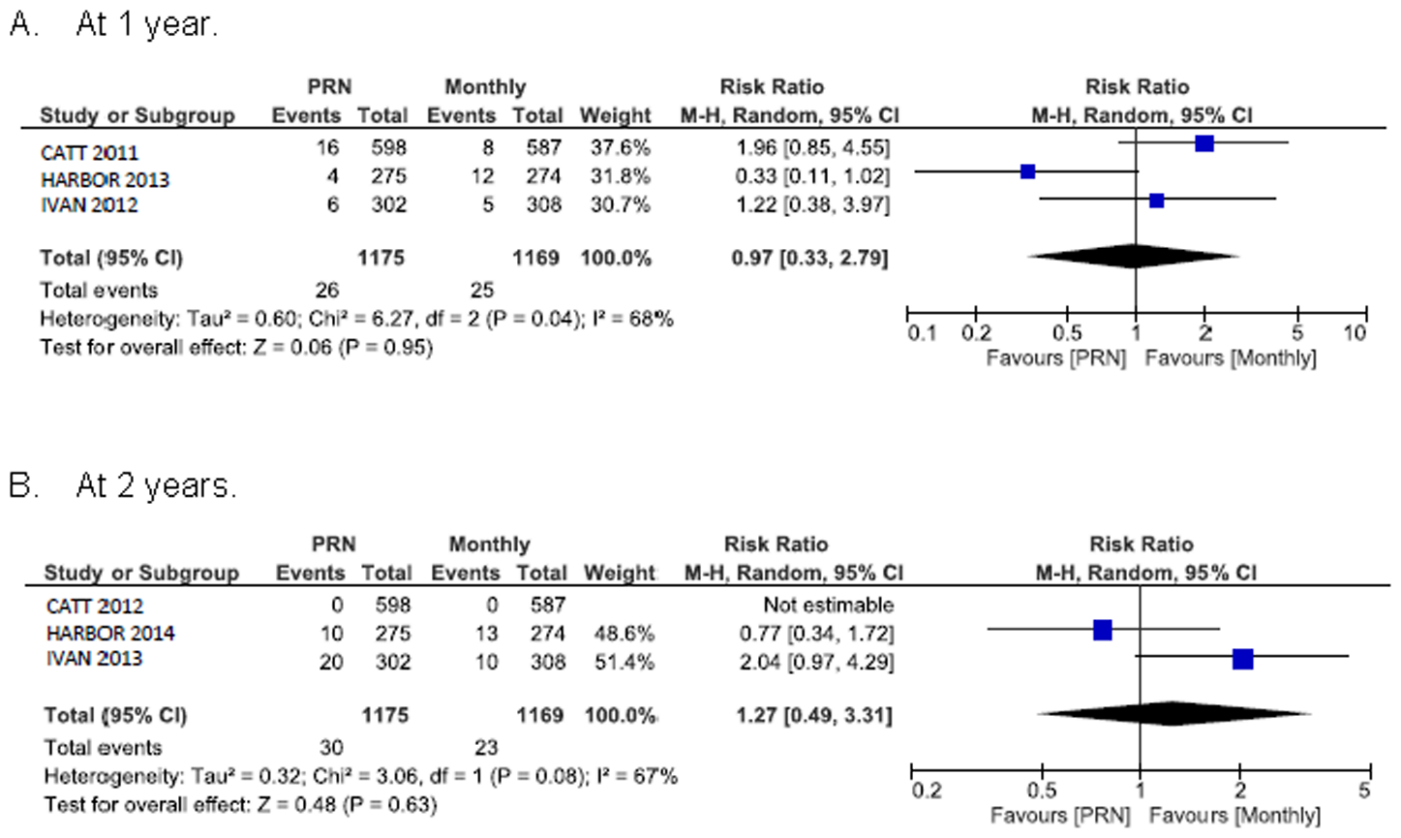
All cause mortality.

**Fig 7:**
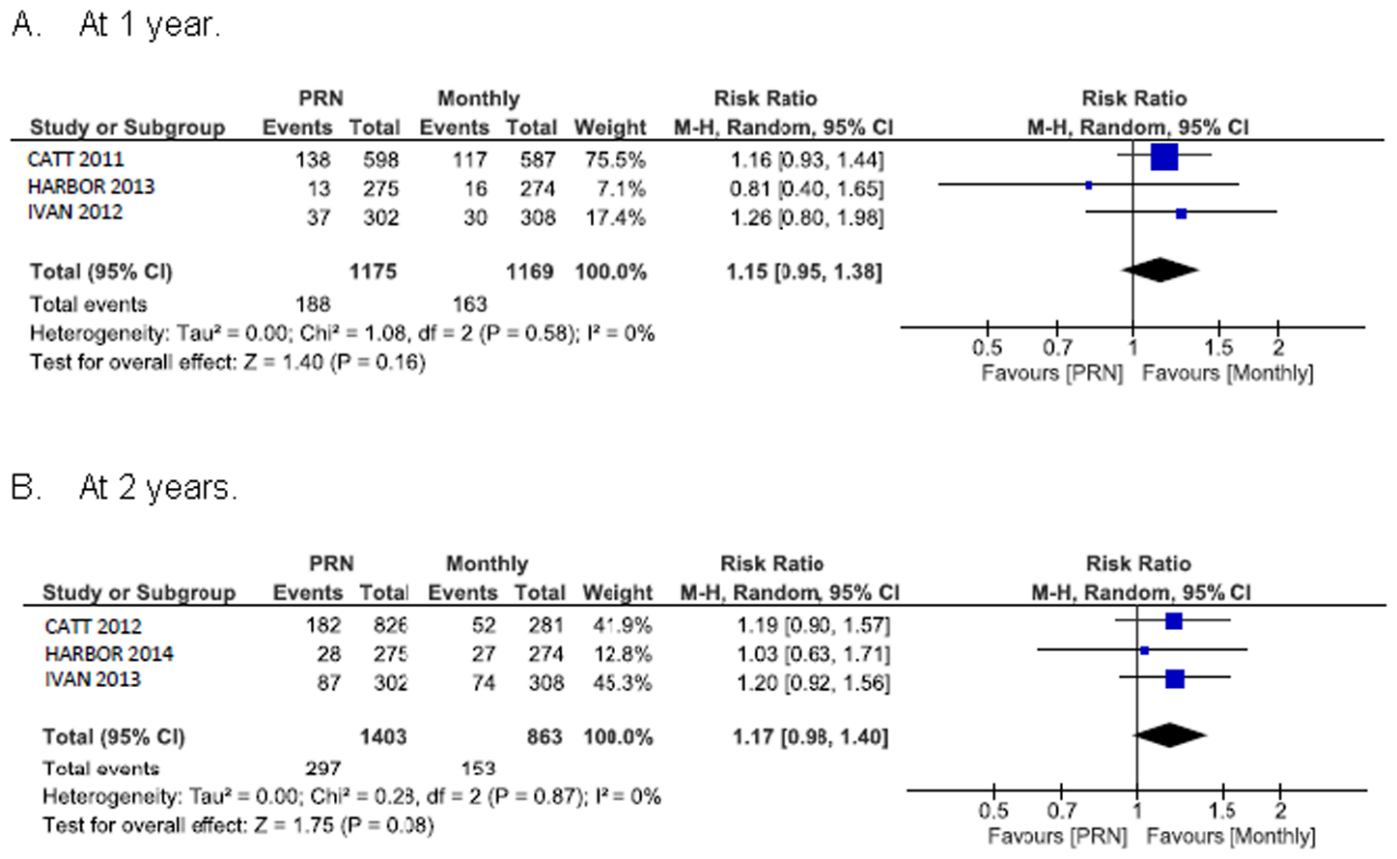
All (more than one) serious systemic adverse event.

**Fig 8:**
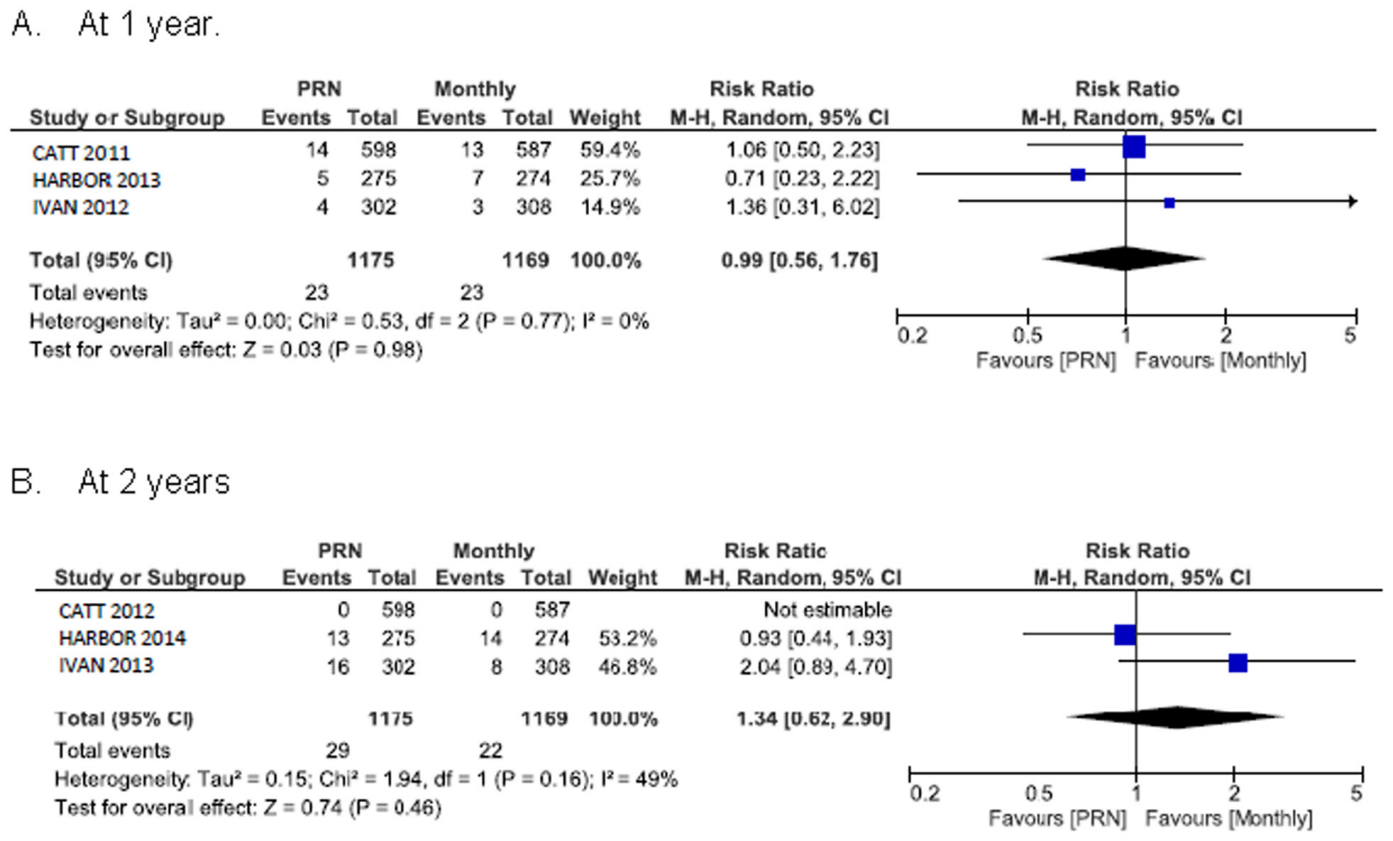
Arterial thromboembolic events.

Fig 6 shows the summary RR for death. The RR of all-cause death for PRN compared with monthly treatment was 1.0 (95% CI 0.3 to 2.8) at 1 year and 1.3 (95% CI 0.5 to 3.3) at 2 years. The 1 year random-effects estimates for the RR of all serious systemic adverse events in patients assigned to PRN versus monthly treatment favoured monthly treatment and were consistent with estimates derived at the end of the longer follow-up (RR 1.2, 95% CI 1.0 to 1.4) (Fig 7). Arterial thromboembolic events were not more frequent under PRN than monthly treatment (RR 1.0, 95% CI 0.6 to 1.8 at 1 year; RR 1.3, 95% CI 0.6 to 2.9 at 2 years) (Fig 8). Using a fixed-effect meta-analysis model on the same studies did not show a statistically significant difference between the treatment regimens. A single head-to-head trial, CATT with a weight between 37.6% and 75.5% (depending on the outcome measure) dominated the meta-analyses.

## Discussion

### Principal findings

The results indicate that an individualized PRN treatment approach with anti-VEGF using visual acuity and OCT-guided retreatment criteria may be appropriate for a wide range of patients with nAMD. Non-inferiority between the treatment regimens was established when using any of the CATT, HARBOR or IVAN non-inferiority margins of 3.5, 4.0, and 5.0 letters, respectively. There were some differences between the trials, for instance, CATT[8, 9] included patients with a Snellen visual acuity between 20/25 and 20/320, HARBOR[12, 13] inclusion was Snellen 20/40-20/320; CATT[8, 9] featured a single loading dose, HARBOR[12, 13] and IVAN[10, 11] had 3 loading doses; patients received 1 injection each time when they met the retreatment criteria in CATT[8, 9] and HARBOR[12, 13] and 3 injections in IVAN[10, 11]; retreatment was guided by SD-OCT in HARBOR, whereas IVAN and CATT were initially controlled by TD-OCT. However, all trials showed that less-than-monthly treatment regimens can decrease treatment burden and still result in visual acuity gains up to 7.9 ETDRS letter over 2 years. In total, the PRN regimen produced 1.9 letters less mean gain than monthly dosing at 2 years. This decrease may be the result of more lesion growth, leakage, and residual fluid on OCT in eyes in the PRN group. Regardless of this minor decrease, the stability and magnitude of the therapeutic effect in the PRN groups is outstanding considering the natural history of nAMD and the moderate efficacy of treatment options before bevacizumab and ranibizumab.

The reported mean gains of visual acuity are the best outcomes observed with less-than-monthly treatment regimens in any long-term, multicentre controlled clinical trial of anti-VEGF drugs. For example, at 2 years, visual acuity has decreased by 2.3 letters after receiving 3 monthly loading doses of ranibizumab 0.5 mg, followed by prescheduled quarterly injections in PIER.[4] In the 2 years, multicentre, extension study SECURE (Long-Term Safety of Ranibizumab 0.5 mg in nAMD) mean BCVA declined by 4.3 letters after ranibizumab was administered if a patient experienced a loss of >5 ETDRS letters measured against the highest visual acuity value obtained.[15] There are several explanations for these differences in visual outcomes. Previous studies had retreatment guidelines that were set according to fixed times, visual acuity decrease and/or morphological changes mainly retinal thickness. The included head-to-head studies, however, applied intravitreal treatment whenever there was evidence of disease activity such as fluid on OCT or bleeding due to nAMD, with no minimum threshold for retinal thickness.[4, 15, 16] The visual outcomes—at least from CATT and HARBOR—were similar to the results observed in the crucial fixed monthly dose trials MARINA and ANCHOR (at 2 years: +5.9 and +7.9 versus +6.6 and +10.7 letters, respectively). During the first 2 years, patients assigned to PRN received a mean of 13.5 injections, which was more than the mean number of injections received in previous studies with a less than monthly treatment strategy.[4, 15]

The meta-analysis of the safety results at 2 years showed that the sum of all serious adverse events differed slightly by treatment regimen. This observation in favour of monthly treatment was also visible when serious adverse events were compared by specific adverse events (mortality and arterial thromboembolic events) at 2 years. There is no clear plausible biological mechanism as yet to explain the slightly increased rates of adverse events with PRN treatment. Therefore, we adhere to the statement of Martin et al 2011[8, 9] that “the difference in rates may be attributable to chance, imbalances in baseline health status that were not included in the medical history or multivariate models, or a true difference in risk.” If there is a true difference in risk, it may be related to idiosyncratic pharmaceutical or disease-related nature. However, to establish whether the ob-served safety signal (RR 1.15) for serious systemic adverse events under PRN compared to monthly treatment is statistically significant, at least 900-something more patients per treatment group would be required.

### Strengths and limitations

Despite the relatively low number of included studies, our evidence is still based on more than 2000 patients followed 2 years. Although RCTs are the best tool to investigate both the efficacy and safety of interventions, they continue to show limitations related to the poor reporting of adverse events, as well as their insufficient power to detect the majority of adverse events, even for common adverse events. Complementary information on the occurrence of adverse events may come from observational studies. However, in this setting, observational studies also show limitations mainly because of poor reporting of adverse events and a lack of direct comparisons between different treatment regimens.[17] For the investigation of safety data, pharmacovigilance remains necessary to explore these issues in the general nAMD population, which often comprises more susceptible patients.

We did not evaluate ocular safety data in this review because many of these, particularly major ocular side events such as sight-threatening bacterial endophthalmitis due to intravitreal injections, are procedure related.[18] Hence, they carry a cumulative risk under more frequent injections—a reason against regular treatment. Due to the fact that both patients and caregivers were aware whether they received an injection or not (lack of double blinding), the evaluation of minor subjective or symptomatic ocular adverse events is associated with a high risk of detection bias leading to an exaggeration of ocular safety concerns.[19] Therefore, we did also not consider these more or less minor subjective events in this review. Generally, absolute rates of serious ocular adverse events are low (≤ 2.1%) and most discordant judgements of anti-VEGF drugs focus on their systemic safety.[17] [20] In addition, the frequency of very rare procedure-related events, such as endophthalmitis, is better studied in large observational series or registries rather that in RCTs.[21]

### Other reviews

A systematic review from Jiang et al 2014 reported on different treatment regimen (monthly versus PRN and quarterly treatment) favouring monthly treatment for visual acuity outcomes.[22] However, this overview is lacking a thorough analysis including event rates, effect estimates and a risk of bias evaluation. Therefore, we are not able to compare our results with this previous research. Chakravarthy et al 2013 pooled 2 year data from the IVAN with 1 year data from the CATT trial and showed a significant difference for deaths favouring monthly treatment.[11] We aggregated these data with a 3^rd^ head-to-head trial (HARBOR) and found no significant differences for this safety issue between the 2 treatment regimens both at 1 and 2 years. Outcomes reported by Chakravarthy et al 2013 on visual acuity and other systemic adverse events such as all serious systemic events and arterial thromboembolic events were comparable with our results. We provided a comprehensive risk of bias assessment targeting safety issues and tested the robustness of the overall estimates by a second analysis—aspects that are lacking in the meta-analysis of Chakravarthy et al. Therefore, as far as we know, this is the first meta-analysis systematically thoroughly addressing PRN treatment versus regular monthly treatment in the management of nAMD.

### Implications for clinical practice

The implications of this review are essential for physicians because the data suggest that PRN treatment based on fluid on OCT and/or visual acuity decline allows for sustained visual improvements over a period of 2 years. However, different issues must be taken into account when considering the PRN approach: (1) Although this regimen decreased treatment burden (frequency) and lead to clinically relevant visual acuity gains, regular visits were still required. (2) In addition, patients in the monthly group performed slightly better on all systemic safety endpoints in comparison to the PRN groups. Therefore, ophthalmologists and patients will need to take into consideration this benefit/risk ratio in the context of decreased injection frequency when deciding to treat with anti-VEGF PRN versus monthly.

## Supporting Information

S1 Excluded Studies Studies Excluded after Full Text Screening with Reasons for Exclusion. (DOCX)

S1 PRISMA Checklist PRISMA 2009 Checklist. (DOC)

S1 Protocol Protocol for a Systematic Review Evaluating Optical Coherence Tomography in Retinal Diseases. (DOCX)

S1 Search Strategy Systematic Literature Search in Medline (via OvidSP). (DOCX)
