# Supplementary material for: Treatment as Required versus Regular Monthly Treatment in the Management of Neovascular Age-Related Macular Degeneration: A Systematic Review and Meta-Analysis
Source: PLoS One. 2015 Sep 14;10(9):e0137866. doi: 10.1371/journal.pone.0137866 (PMC4569266; doi:10.1371/journal.pone.0137866)
Supplement: S1 Excluded Studies — (DOCX) [file pone.0137866.s001.docx]

**Figure S3.** Studies excluded after full text screening with reasons for exclusion.

**Reason for exclusion: treatment schema not relevant (N=10)**

Abraham P, Yue H, Wilson L. Randomized, double-masked, sham-controlled trial of ranibizumab for neovascular age-related macular degeneration: PIER study year 2. Am J Ophthalmol 2010;150(3):315-24.e1.

Barikian A, Mahfoud Z, Abdulaal M, et al. Induction with intravitreal bevacizumab every two weeks in the management of neovascular age-related macular degeneration. Am J Ophthalmol 2015;159(1):131-7.

Kodjikian L, Souied EH, Mimoun G, et al. Ranibizumab versus Bevacizumab for Neovascular Age-related Macular Degeneration: Results from the GEFAL Noninferiority Randomized Trial. Ophthalmology 2013;120(11):2300-9.

Li X, Hu Y, Sun X, et al. Bevacizumab for neovascular age-related macular degeneration in China. Ophthalmology 2012;119(10):2087-93.

Lushchyk T, Amarakoon S, Martinez-Ciriano JP, et al. Bevacizumab in age-related macular degeneration: a randomized controlled trial on the effect of injections every 4weeks, 6weeks and 8weeks. Acta Ophthalmol (Oxf) 2013;91(6):e456-61.

Regillo CD, Brown DM, Abraham P, et al. Randomized, double-masked, sham-controlled trial of ranibizumab for neovascular age-related macular degeneration: PIER Study year 1. Am J Ophthalmol 2008;145(2):239-48.

Schmidt-Erfurth U, Eldem B, Guymer R, et al. Efficacy and safety of monthly versus quarterly ranibizumab treatment in neovascular age-related macular degeneration: the EXCITE study. Ophthalmology 2011;118(5):831-9.

Tufail A, Patel PJ, Egan C, et al. Bevacizumab for neovascular age related macular degeneration (ABC Trial): multicentre randomised double masked study. BMJ 2010;340:c2459.

Wykoff CC, Brown DM, Chen E, et al. SAVE (Super-dose anti-VEGF) trial: 2.0 mg ranibizumab for recalcitrant neovascular age-related macular degeneration: 1-year results. Ophthalmic Surg Lasers Imaging Retina 2013;44(2):121-6.

Wykoff CC, Brown DM, Croft DE, et al. Two Year SAVE Outcomes: 2.0 mg ranibizumab for recalcitrant neovascular AMD. Ophthalmology 2013;120(9):1945-6.e1.

**Reason for exclusion: study design (non-RCTs or case series; N=17)**

Non-RCTs:

Gupta B, Adewoyin T, Patel SK, et al. Comparison of two intravitreal ranibizumab treatment schedules for neovascular age-related macular degeneration. Br J Ophthalmol 2011;95(3):386-90.

Oubraham H, Cohen SY, Samimi S, et al. Inject and extend dosing versus dosing as needed: a comparative retrospective study of ranibizumab in exudative age-related macular degeneration. Retina 2011;31(1):26-30.

Krebs I, Binder S, Stolba U, et al. Optical coherence tomography guided retreatment of photodynamic therapy. Br J Ophthalmol 2005;89(9):1184-7.

Case series:

Abedi F, Wickremasinghe S, Islam AF, et al. Anti-VEGF treatment in neovascular age-related macular degeneration: a treat-and-extend protocol over 2 years. Retina 2014;34(8):1531-8.

Casaroli-Marano R, Gallego-Pinazo R, Fernandez-Blanco CT, et al. Age-Related Macular Degeneration: Clinical Findings following Treatment with Antiangiogenic Drugs. J Ophthalmol 2014;2014:346360.

Chen CY, Wong TY, Heriot WJ. Intravitreal bevacizumab (Avastin) for neovascular age-related macular degeneration: a short-term study. Am J Ophthalmol 2007;143(3):510-2.

Cohen SY, Dubois L, Tadayoni R, et al.: Results of one-year's treatment with ranibizumab for exudative age-related macular degeneration in a clinical setting. Am J Ophthalmol 2009;148(3):409-13.

Cohen SY, Mimoun G, Oubraham H, et al. Changes in visual acuity in patients with wet age-related macular degeneration treated with intravitreal ranibizumab in daily clinical practice: the LUMIERE study. Retina 2013;33(3):474-81.

Fung AE, Lalwani GA, Rosenfeld PJ, et al. An optical coherence tomography-guided, variable dosing regimen with intravitreal ranibizumab (Lucentis) for neovascular age-related macular degeneration. Am J Ophthalmol 2007;143(4):566-83.

Gillies MC, Walton R, Simpson JM, et al. Prospective audit of exudative age-related macular degeneration: 12-month outcomes in treatment-naive eyes. Invest Ophthalmol Vis Sci 2013;54(8):5754-60.

Gupta OP, Shienbaum G, Patel AH, et al. A treat and extend regimen using ranibizumab for neovascular age-related macular degeneration clinical and economic impact. Ophthalmology 2010;117(11):2134-40.

Holz FG, Amoaku W, Donate J, et al. Safety and efficacy of a flexible dosing regimen of ranibizumab in neovascular age-related macular degeneration: the SUSTAIN study. Ophthalmology 2011;118(4):663-71.

Inoue M, Arakawa A, Yamane S, et al. Intravitreal injection of ranibizumab using a pro re nata regimen for age-related macular degeneration and vision-related quality of life. Clin 2014;8:1711-6.

Lalwani GA, Rosenfeld PJ, Fung AE, et al. A variable-dosing regimen with intravitreal ranibizumab for neovascular age-related macular degeneration: year 2 of the PrONTO Study. Am J Ophthalmol 2009;148(1):43-58.e1.

Menke MN, Zinkernagel MS, Ebneter A, et al. Functional and anatomical outcome of eyes with neovascular age-related macular degeneration treated with intravitreal ranibizumab following an exit strategy regimen. Br J Ophthalmol 2014;98(9):1197-200.

Silva R, Axer-Siegel R, Eldem B, et al. The SECURE study (non-RCT): long-term safety of ranibizumab 0.5 mg in neovascular age-related macular degeneration. Ophthalmology 2013;120(1):130-9.

Wolf A, Kampik A. Efficacy of treatment with ranibizumab in patients with wet age-related macular degeneration in routine clinical care: data from the COMPASS health services research. Graefes Arch Clin Exp Ophthalmol 2014;252(4):647-55.
