# Supplementary material for: Treatment as Required versus Regular Monthly Treatment in the Management of Neovascular Age-Related Macular Degeneration: A Systematic Review and Meta-Analysis
Source: PLoS One. 2015 Sep 14;10(9):e0137866. doi: 10.1371/journal.pone.0137866 (PMC4569266; doi:10.1371/journal.pone.0137866)
Supplement: S1 Protocol — (DOCX) [file pone.0137866.s003.docx]

**Figure S1.** Protocol for a systematic review evaluating optical coherence tomography in retinal diseases.

***‚OCT-Verfahren in der Indikation feuchte altersabhängige Makuladegeneration und diabetische Retinopathie‘***

Deutsches Cochrane Zentrum

Institut für Med. Biometrie u. Med. Informatik

Universitätsklinikum Freiburg

Berliner Allee 29

79110 Freiburg (Germany)

Email: schmucker@cochrane.de

Tel.: 0761/203-6695 (Fax:-6712)

**Inhaltsverzeichnis**

[**Abkürzungsverzeichnis** 3](#_Toc427051931)

[1. Altersabhängige Makuladegeneration 4](#_Toc427051932)

[1.1 Indikation 4](#_Toc427051933)

[1.2 Interventionen 4](#_Toc427051934)

[1.3 Diagnostische Verfahren 5](#_Toc427051935)

[2. Diabetische Retinopathie 5](#_Toc427051936)

[2.1 Indikation 5](#_Toc427051937)

[2.2 Interventionen 6](#_Toc427051938)

[2.3 Diagnostische Verfahren 6](#_Toc427051939)

[3. Problembeschreibung / Rationale 7](#_Toc427051940)

[4. Ziele der Systematischen Übersichtsarbeit 7](#_Toc427051941)

[5. Methodische Vorgehensweise 9](#_Toc427051942)

[5.1 Entwicklung der methodischen Ansätze zur Klärung der Fragestellungen 9](#_Toc427051943)

[5.1.1 Ziel 1: Studien, die die diagnostische Genauigkeit des OCT-Verfahren evaluieren 9](#_Toc427051944)

[5.2.2 Ziel 2: Studien, die das OCT-Verfahren zur Therapiesteuerung evaluieren 9](#_Toc427051945)

[5.2 Informationsbeschaffung 12](#_Toc427051946)

[5.3 Extraktion der Studiendaten und Informationsbewertung 12](#_Toc427051947)

[5.4 Informationssynthese und –analyse 13](#_Toc427051948)

[6. Literatur 15](#_Toc427051949)

**Abkürzungsverzeichnis**

| **Abkürzung** | **Bedeutung** |
| --- | --- |
| AMD | Altersabhängige Makuladegeneration |
| CNV | Chorioidale Neovaskularisation |
| DMÖ | Diabetisches Makulaödem |
| DR | Diabetische Retinopathie |
| EMA | European Medicines Agency |
| Embase | Excerpta Medica |
| ETDRS | Early Treatment of Diabetes Retinopathy Study |
| FA | Fluoreszeinangiographie |
| OCT | Optische Kohärenztomographie |
| PlGF | Plazenta-Wachstumsfaktor |
| RCT | Randomisierte kontrollierte Studie |
| VEGF | Vaskuläre endotheliale Wachstumsfaktoren |
| WHO | World Health Organisation |

# Altersabhängige Makuladegeneration

## 1.1 Indikation

In Deutschland ist die altersabhängige Makuladegeneration (AMD) mit etwa 50% die häufigste Ursache für schwere Sehbehinderungen ([1](#_ENREF_1)). Weltweit sind etwa 30 Millionen Menschen, in Deutschland etwa 4,5 Millionen Menschen, von dieser Krankheit betroffen ([2](#_ENREF_2)). Bei der AMD werden zwei späte Verlaufsformen, die „feuchte“ und die „trockene“ AMD Form, unterschieden. Die trockene Verlaufsform, an der in etwa 85% bis 90% der Patienten erkranken, ist in der Regel durch einen langsamen Verlust der Sehkraft gekennzeichnet ([3](#_ENREF_3)). An der feuchten (neovaskuläre oder exsudative) AMD Form erkranken zwischen 10% und 15% der Patienten ([4](#_ENREF_4)). Diese Verlaufsform führt jedoch in über 90% der Fälle zu schweren Sehbeeinträchtigungen ([5](#_ENREF_5)). Morphologisch ist die feuchte Verlaufsform durch eine pathologische Einsprossung neuer (brüchiger) Blutgefäße, die von der Aderhaut ausgehen und in den subretinalen Raum vordringen, gekennzeichnet. Die Bildung neuer Gefäße wird als chorioidale Neovaskularisation (CNV) bezeichnet.

## 1.2 Interventionen

In den letzten Jahrzehnten standen für die Behandlung der feuchten AMD medikamentöse Interventionen (z.B. die Photodynamische Therapie, Kortikosteroidpräparate [z.B. Triamcinolon], VEGF-Antikörper) und nicht-medikamentöse Interventionen (z.B. Laserkoagulation, Radiotherapie, Thermotherapie) zur Verfügung. Da es bei der feuchten AMD zum Wachstum krankhaft veränderter Aderhautgefäße im Bereich der Makula kommt (siehe Abschnitt 1.1), nimmt seit etwa dem Jahr 2005 die Hemmung von vaskulären endothelialen Wachstumsfaktoren (VEGF) durch VEGF-Antikörper wie Ranibizumab (Lucentis®), Bevacizumab (Avastin®) oder Pegaptanib (Macugen®) eine zentrale Stellung bei der AMD Behandlung ein ([6](#_ENREF_6),7). Neu zugelassen wurde im Dezember 2012 auch das antikörperähnliche, rekombinante VEGF-Rezeptorprotein Aflibercept (VEGF Trap, Eylea®), welches neben VEGF auch den Plazenta-Wachstumsfaktor (PIGF) bindet (8). Die hohe Wirksamkeit der VEGF-Antikörper wird bei der AMD durch eine initiale Behandlung mit drei intravitrealen Injektionen in monatlichen Abständen erzielt. Danach wird eine individualisierte Therapie bei fortbestehender Aktivität der CNV nach einem pro-re-nata (nach Bedarf) Schema empfohlen (9). Die Häufigkeit und diagnostische Gestaltung der Erhaltungskontrollen sind dabei für den langfristigen Therapieerfolg bei der Behandlung der AMD entscheidend (9).

## 1.3 Diagnostische Verfahren

Den diagnostischen Goldstandard zur Diagnose der AMD stellt die Fluoreszeinangiographie (FA) dar (9). Bei der FA wird ein fluoreszierender Farbstoff intravenös verabreicht und dessen Verteilung in den Gefäßen des Auges beobachtet. Die FA ist die Grundlage für die Einteilung der bei der feuchten AMD auftretenden CNV (klassische oder okkulte CNV). Neben der FA erfolgen bei der Grunddiagnostik auch eine Erhebung der bestkorrigierten Sehschärfe (Visus) und eine Funduskopie (9).

Im Gegensatz zur FA ist die optische Kohärenztomographie (OCT) ein nicht-invasives hochauflösendes bildgebendes Verfahren, das die verschiedenen Strukturen der Netzhaut abbildet. Es ermöglicht somit die Beurteilung intra- und subretinaler Strukturen, wie z.B. Flüssigkeitsansammlungen, Narbenprozesse und der Netzhautdicke. Die OCT wird als (additive) Untersuchung bei der Diagnosestellung mit dem Hintergrund eingesetzt, die Ausgangssituation für den Vergleich der Therapieeffekte im Verlauf der AMD zu dokumentieren. Neben der bildgebenden Therapiesteuerung mit dem OCT-Verfahren kann auch der Verlauf der subjektiv erhobenen Sehschärfe und die Wahrnehmung verzerrter Seheindrücke als funktionelles Kriterium für die Wiederbehandlung herangezogen werden (9).

# Diabetische Retinopathie

## 2.1 Indikation

Die diabetische Retinopathie (DR) ist die häufigste Ursache für eine Erblindung der Menschen im berufsfähigen Alter. Nahezu alle Patienten mit Typ-1 Diabetes und in etwa 60% der Patienten mit Typ-2 Diabetes erkranken im Laufe ihres Lebens an dieser Augenerkrankung (10). Bei der DR unterscheidet man zwischen der nichtproliferativen und proliferativen Form: Die nicht[proliferative](http://de.wikipedia.org/wiki/Zellproliferation) Retinopathie verläuft zunächst asymptomatisch. Auf der Netzhaut sind zwar krankhafte Veränderungen wie z.B. Mikroaneurysmen und retinale Blutungen sichtbar, krankhafte Gefäßneubildungen sind jedoch bei der nichtproliferativen Form noch nicht vorhanden. Rund 50% der Patienten mit einer nichtproliferativen Retinopathie entwickeln in relativ kurzer Zeit (innerhalb eines Jahres) durch eine zunehmende Freisetzung von Wachstumsfaktoren, wie z.B. VEGF, eine proliferative Form (11). Die proliferative Form geht dabei mit der Neubildung von krankhaften Blutgefäßen einher. Diese abnormalen Gefäße können zu Blutungen führen oder die Netzhaut vom Untergrund abheben (traktive Netzhautablösung), was zur Erblindung führen kann.

Das *diabetische Makulaödem (DMÖ)* ist die häufigste Ursache für eine Sehverschlechterung bei der DR und kann in jedem Stadion der DR auftreten. Das DMÖ wird seit der Early Treatment of Diabetes Retinopathy Study (ETDRS) als eine Netzhautverdickung oder das Vorhandensein harter Exsudate innerhalb eines Papillendurchmessers von der Fovea definiert (12). Das klinisch signifikante Makulaödem stellt dabei die schwerwiegendste Form dar und ist über die Beteiligung der Fovea definiert.

## 2.2 Interventionen

Die Lasertherapie galt bis vor kurzem als Mittel der ersten Wahl bei der Behandlung des DMÖ. Sie hat sich als effektive Maßnahme zur Stabilisierung der Sehschärfe etabliert, die Wahrscheinlichkeit eine Verbesserung der Sehkraft zu erfahren ist dabei aber gering (13,14). Neben der Lasertherapie kamen in der Vergangenheit auch intravitreale Injektionen von Kortikosteroiden zum Einsatz, die aber durch ihr Nebenwirkungsprofil (vor allem Sekundärglaukom und Sekundärkatarakt) als Therapieoption eher in den Hintergrund getreten sind (15). Durch die Einführung der VEGF-Antikörper haben sich, ähnlich wie bei der Behandlung der AMD, erfolgsversprechende Therapieoptionen der DR, vor allem im Zusammenhang mit einem DMÖ, ergeben. Die VEGF-Antikörper werden dabei zu Beginn der Therapie in regelmäßigem Abstand direkt ins Auge injiziert - solange bis eine Verbesserung des klinischen Befunds eingetreten ist. Danach wird der Antikörper nur noch dann injiziert, wenn im Zuge der Nachkontrolle eine Verschlechterung des klinischen Befunds bzw. der Sehkraft festgestellt wird (pro-re-nata Schema). Im Gegensatz zur AMD zieht sich der Visusanstieg beim DMÖ nach Therapiebeginn in der Regel über einen längeren Zeitraum hin (16).

## 2.3 Diagnostische Verfahren

Bei der diabetischen Retinopathie erfolgt die Indikationsstellung mit der stereoskopischen Fundusuntersuchung (biomikroskopische Untersuchung der Netzhaut in Mydriasis) und Prüfung der Sehschärfe (16). Mit der Funduskopie können in erster Linie Blutungen oder Mikroaneurysmen in der Makula diagnostiziert werden, eine Messung der Netzhautdicke ist jedoch nicht direkt möglich. Neben der stereoskopischen Fundusuntersuchung wird zur Indikationsstellung auch die FA empfohlen, um zentrale Störungen der Blut-Retina-Schranke und eine eventuelle Ischämie sowie Ursprung und Ausdehnung der Neovaskularisation beurteilen zu können. Zusätzlich stellt das OCT-Verfahren eine wesentliche diagnostische Maßnahme bei der DR dar (16). Die hochauflösende Spectral Domain OCT kann dabei wichtige Informationen im Hinblick auf die Quantifizierung der makulären Netzhautdicke und der qualitativen Beurteilung verschiedener Netzhautschichten liefern, die für die Beurteilung der DR (vor allem im Hinblick auf das DMÖ) im Verlauf von Bedeutung sein können (16). Im Gegensatz zur subjektiven stereoskopischen Funduskopie und der nicht quantifizierbaren Leckage bei der FA stellt das OCT-Verfahren eine objektive diagnostische Maßnahme dar.

# Problembeschreibung / Rationale

Die OCT kommt immer häufiger als routinemäßiges objektives diagnostisches Verfahren in der Indikation feuchte AMD und DR zum Einsatz. Jedoch ist die diagnostische Genauigkeit des OCT-Verfahrens gegenüber dem Goldstandard bzw. gegenüber herkömmlicher diagnostischer Verfahren bei beiden Indikationen nicht ausreichend geklärt. Es besteht außerdem Klärungsbedarf, ob die in der klinischen Praxis in erster Linie durchgeführte Therapiesteuerung durch OCT (morphologische Verlaufsbeurteilung) gegenüber der herkömmlichen Therapiesteuerung durch funktionelle Parameter (Beurteilung der Sehschärfe) einen Zusatznutzen für AMD bzw. DR Patienten im Hinblick auf den Erhalt der Sehschärfe, unerwünschte Wirkungen und Lebensqualität aufweist. In der ophthalmologischen Stellungnahme zur AMD wird zwar ‚Morphologie‘ vor ‚Funktion‘ postuliert, jedoch bezieht sich die Evidenzlage für diese Handlungsempfehlung in erster Linie auf Studientypen, die mit einem hohen Verzerrungspotential in Zusammenhang gebracht werden (wie zum Beispiel Fallserien [17]).

# Ziele der Systematischen Übersichtsarbeit

Ziel der geplanten Systematischen Übersichtsarbeit ist es deshalb, die diagnostische Genauigkeit des OCT-Verfahrens (Indextest) im Vergleich zum jeweiligen Referenzstandard (bei der Indikation AMD und DR) zur Diagnosesicherung zu evaluieren. Außerdem soll geklärt werden, ob das OCT-Verfahren einen Zusatznutzen bei der Therapiesteuerung gegenüber diagnostischen Verfahren, die funktionelle Parameter (d.h. die Sehschärfe) messen, aufweist.

Im Detail soll das *OCT-Verfahren* dabei in Hinblick auf folgende Ziele und Zielgrößen bewertet werden:

- **Ziel 1: OCT zur Diagnosesicherung**
- bei Ersterkrankung
- zur Indikation der Behandlungsinitiierung
- zur Beurteilung des Therapieerfolgs

Zielgrößen sind dabei diagnostische Gütekriterien (wie z.B. Sensitivität/Spezifität, Likelihood Ratios, diagnostische Odds Ratio) und der positive und negative Vorhersagewert des Untersuchungsverfahrens.

- **Ziel 2: OCT zur Therapiesteuerung**

Zielgrößen sind dabei patientenrelevante Endpunkte, z.B. der Therapieerfolg (=Erhalt oder Verbesserung der Sehschärfe, geringere Anzahl an Injektionen bei gleichem Therapieerfolg), Zusatzbelastungen für den Patienten (=höhere Anzahl an Injektionen und ein damit verbundenes höheres Sicherheitsrisiko, Zusatzuntersuchungen, z.B. durch FA, und ein damit verbundenes höheres Sicherheitsrisiko) und Lebensqualität.

Um diesen Zielen ausreichend Rechenschaft zu tragen, werden folgende Aspekte in der Systematischen Übersichtsarbeit besondere Berücksichtigung finden:

- Definition der Krankheitsbilder einschließlich Epidemiologie, für die das OCT-Verfahren als diagnostische Maßnahme oder zur Therapiesteuerung eingesetzt wird.
- Definition der Therapien, für die das OCT-Verfahren zur Steuerung des Behandlungserfolgs eingesetzt wird.
- Definition des jeweiligen Referenzstandards (für die Indikation AMD und DR) und der herkömmlichen Messverfahren, mit denen das OCT-Verfahren zur Diagnosesicherung und Therapiesteuerung verglichen werden soll.
- Definition der Messparameter/Endpunkte und Schwellenwerte zur Diagnosestellung und Therapieinitiierung der jeweiligen diagnostischen Verfahren:
- *OCT*: morphologische Parameter, wie z.B. Netzhautdicke
- *FA*: morphologische Parameter, wie z.B. Größe der Läsion
- *Funduskopie*: morphologische Parameter, wie z.B. Aneurysma
- *Sehtes*t: funktionelle Parameter, wie z.B. ETDRS-Sehschärfe

# Methodische Vorgehensweise

## 5.1 Entwicklung der methodischen Ansätze zur Klärung der Fragestellungen

### 5.1.1 Ziel 1: Studien, die die diagnostische Genauigkeit des OCT-Verfahren evaluieren

Diese Fragestellung lässt sich durch Diagnosestudien beantworten, die die diagnostische Genauigkeit des OCT-Verfahren zur 1) Diagnosestellung bei Ersterkrankung, 2) Indikation der Behandlungsinitiierung und 3) Beurteilung des Therapieerfolgs im Vergleich zu einem Referenztest evaluieren. Die Diagnosestudien müssen dabei Angaben zu diagnostischen Gütekriterien (z.B. Sensitivität, Spezifität) bzw. zu Angaben aus denen eine Ableitung von Gütekriterien erfolgen kann (z.B. Vierfeldertafel) und/oder Vorhersagewerten machen.

Ein- und Ausschlusskriterien sollen dabei für folgende Parameter definiert werden:

- Indikation/Krankheitsbild (Welche Patientenpopulation wird eingeschlossen? – d.h. Alter der eingeschlossenen Patienten, Komorbidität, Vorbehandlung, bei AMD Patienten: CNV-Typ, bei DR Patienten: Ausprägung des DMÖ)
- Indextext (Welcher Indextest kommt zum Einsatz? - z.B. nur bestimmte OCT-Typen, oder jegliche Modelle?)
- Referenztest (Welche Referenzverfahren sind zulässig? - z.B. nur der Goldstandard (FA) oder jegliches diagnostische Verfahren?)
- Zielgrößen (Welche Zielgrößen werden evaluiert? - d.h. Angaben zu diagnostischen Gütekriterien (Sensitivität, Spezifität) und/oder Vorhersagewerten)
- Studientypen (Welche Studientypen ziehe ich zur Beantwortung der Fragestellung heran? - z.B. jeglicher Studientyp [nach Absprache mit Auftraggeber]?)

### 5.2.2 Ziel 2: Studien, die das OCT-Verfahren zur Therapiesteuerung evaluieren

Diese Fragestellung lässt sich durch Interventionsstudien beantworten, in denen das OCT-Verfahren zur Steuerung des Therapieerfolgs eingesetzt wurde. Die Intervention stellt dabei das OCT-Verfahren dar, als *Komparator* dient der Sehtest (sowohl bei der Indikation AMD als auch bei der Indikation DR/DMÖ) ± evtl. zusätzliche diagnostische Verfahren (z.B. Funduskopie). Folgende Ansätze scheinen zur Bearbeitung der Fragestellung sinnvoll:

OCT *vs* Sehtest:

Direkte Vergleiche (innerhalb einer Studie)

- Die beste verfügbare Evidenz liefern randomisierte kontrollierte Interventionsstudien, in denen der Vergleich OCT *vs* Sehtest im Hinblick auf patientenrelevante Endpunkte durchgeführt wird.
- Sollte die aktuelle Studienlage keine direkten Vergleiche durch randomisierter Patientenzuordnung liefern, können auch vergleichende nicht-randomisierte Interventionsstudien zur Beantwortung der Fragestellung herangezogen werden.

Indirekte Vergleiche (zwischen verschiedenen Studien)

- Sollte die Studienlage, die direkte Vergleiche innerhalb randomisierter und nicht-randomisierter Interventionsstudien durchführt, unbefriedigend sein, können indirekte Vergleiche herangezogen werden. Dabei werden die Endpunkte verschiedener Studien mit unterschiedlicher Therapiesteuerung indirekt miteinander verglichen. Indirekte Vergleiche auf Grundlage von randomisierten kontrollierten Studien, die einen gemeinsamen Komparator aufweisen (z.B. Studie 1: OCT *vs* Vergleichstest C; Studie 2: Sehschärfemessung *vs* Vergleichstest C), können dabei als valider betrachtet werden als indirekte Vergleiche basierend auf Beobachtungsstudien (z.B. Fallserien), bei denen nur die entsprechenden Studienarme verglichen werden können (z.B. Studie 1: OCT-Verfahren; Studie 2: Sehschärfemessung).
- Um indirekte Vergleiche durchzuführen, müssen die Studien folgende Voraussetzung erfüllen: identisches Medikament (z.B. Dosierung, Applikationsmethode), vergleichbarer Abstand der Nachuntersuchungstermine, vergleichbare Patientenkollektive (z.B. Alter, Krankheitsstadium), vergleichbare Zielgrößenerhebung (z.B. Nachbeobachtungszeit, Messmethode der Zielgrößenerhebung).

Pro-re-nata Behandlung (OCT gesteuerte) vs monatliche Behandlung

- Hintergrund für diesen Ansatz: In den Zulassungsstudien zu Ranibizumab wurde monatlich injiziert, da man davon ausging, dass die funktionellen Parameter damit aufrechterhalten bzw. verbessert werden können (z.B. 18,19). In den ‚neueren‘ Studien werden die VEGF-Antikörper pro-re-nata injiziert; d.h. das Wiederbehandlungskriterium wird an morphologischen Veränderungen mit Hilfe des OCT-Verfahrens gemessen (Morphologie vor Funktion) (z.B. 20,21).
- Die zuverlässigsten Daten zur Evaluierung dieses Ansatzes liefern wieder direkt vergleichende Studien; d.h. Studien, in denen die pro-re-nata Strategie mit der monatlichen Behandlung direkt verglichen wird (siehe oben).
- Falls direkte Vergleiche nicht wie im gehofften Umfang zur Verfügung stehen sollten, kämen auch bei diesem Ansatz indirekte Vergleiche in Frage. D.h., vergleicht man die Endpunkte der Studien, in denen monatlich injiziert wird mit den Endpunkten der Studien, in denen pro-re-nata injiziert wird (OCT gesteuerte Studien), sollte es möglich sein, indirekt den Nutzen des OCT-Verfahrens auf den Therapieerfolg zu bewerten. Voraussetzung: identisches Medikament, vergleichbare Patientenkollektive (z.B. Alter, Krankheitsstadium), vergleichbare Zielgrößenerhebung (z.B. Nachbeobachtungszeit, Messmethode der Zielgrößenerhebung).

Ein- und Ausschlusskriterien sollen dabei für folgende Parameter definiert werden:

- Indikation/Krankheitsbild (z.B. welche Patientenpopulation wird eingeschlossen? – d.h. Alter der eingeschlossenen Patienten, Komorbidität, Vorbehandlung, bei AMD Patienten: CNV-Typen, bei DR Patienten: Ausprägung des DMÖ)
- Intervention: OCT-Verfahren zur Therapiesteuerung (Besteht die Intervention nur aus dem OCT-Verfahren, oder sind auch Kombinationen, wie z.B. OCT + Sehtest + FA, wie es bei der CATT Studie (20) zur Therapiesteuerung erfolgte, zulässig? => Indextest nicht vollständig unabhängig von Referenztest!)
- Komparator: Referenztest (Welcher Referenztest ist zulässig? - nur Messverfahren um funktionelle Parameter zu messen oder auch Kombination mit morphologischen Messverfahren?)
- Zielgrößen (Welche Endpunkte muss die Studie berichten? – d.h. patientenrelevante Endpunkte wie Erhalt/Verbesserung der Sehschärfe, unerwünschte Wirkungen, Lebensqualität)
- Nachbeobachtungszeit (Sollen die eingeschlossen Studien eine bestimmte Nachbeobachtungszeit aufweisen?)
- Studientypen (Welche Studientypen sollen evaluiert werden? - z.B. randomisierte kontrollierte Studien, Beobachtungsstudien wie z.B. Fallserien [nach Absprache mit Auftraggeber])

## 5.2 Informationsbeschaffung

Ziel der Informationsbeschaffung wird es sein, publizierte und nicht publizierte Studien zu identifizieren, die zur Frage der diagnostischen Genauigkeit für die Indikationsstellung (Diagnosestudien) und zur Frage der Therapieentscheidung durch das OCT-Verfahren (Interventionsstudien) wesentliche Informationen liefern.

Die Literatursuche nach relevanten, veröffentlichten Studien nach den Methoden des Cochrane Handbuches (17) wird mit Unterstützung einer Expertin in der Literaturrecherche entwickelt. Folgende elektronische Datenbanken sollen dabei durchsucht werden: Medline, Embase, Cochrane Library, Cinahl, Psyndex, PsycINFO, Social Sci Search, Web of Science. Nach momentan laufenden Studien wird im Register für klinische Studien (ClinicalTrials.gov) und in der World Health Organisation (WHO) International Clinical Trials Registry Platform (<http://www.who.int/ictrp/en/>) gesucht.

Nach Abschluss der Literatursuche werden die bibliographischen Angaben der identifizierten Publikationen zum Zwecke der weiteren Bearbeitung in ein Literaturverwaltungsprogramm (Endnote) importiert.

In einem ersten Auswahlschritt werden die identifizierten Literaturzitate anhand ihres Titels und – sofern vorhanden – anhand ihres Abstracts von zwei Reviewern unabhängig voneinander gesichtet, um zu entscheiden, welche hiervon unter Beachtung der vordefinierten Ein- und Ausschlusskriterien von beiden Reviewern als sicher relevant eingeordnet werden können. Studien, die dabei als potenziell relevant eingestuft werden, werden im Volltext gelesen (Volltextscreening, Entscheidung über Ein- oder Ausschluss anhand des Volltextes).

## 5.3 Extraktion der Studiendaten und Informationsbewertung

Dieser Schritt beinhaltet die Erstellung der Datenextraktionsbögen und die Datenextraktion der eingeschlossenen Studien. Um eine hohe Ergebnissicherheit zu erzielen, wird die Datenextraktion ebenfalls von zwei unabhängigen Reviewern ausgeführt.

Für die Qualitätsbewertung der **Diagnosestudien** wird das Instrument QUADAS-2 (Quality of Diagnostic Accuracy Studies) verwendet (22). Dieses Qualitätsbewertungsinstrument besteht aus 14 Fragen, wie z.B. Generalisierbarkeit (im Sinne der Übertragbarkeit der Testergebnisse auf die Anwendung im klinischen Alltag), Informationen zu Genauigkeit und Unabhängigkeit des Referenztests (Vergleichstest) vom zu prüfenden Test (Indextest: OCT), verblindete Interpretation der Testergebnisse, Darstellung nicht interpretierbarer Testergebnisse und Erläuterung von Studienabbrechern.

Die Qualität der **Interventionsstudien,** die das OCT-Verfahren zur Therapiesteuerung evaluieren, wird nach den Methoden des Cochrane Handbuchs für randomisierte und nicht-randomisierte Studien getrennt bewertet (17). Für randomisierte Studien werden dabei Aspekte wie z.B. Generierung der Randomisierungssequenz, Concealment of Allocation, Verblindung, Intention-to-treat Analyse und Nachbeobachtungszeit ausgewertet. Für nicht-randomisierte Studien werden dagegen Kriterien wie z.B. die Repräsentativität der Stichprobe, Krankheitsstadium zu Behandlungsbeginn und Nachbeobachtungszeit von Bedeutung sein.

Unter Berücksichtigung der methodischen Aspekte wird es möglich sein, das Verzerrungsrisiko (d.h. Einteilung der einzelnen Studien nach z.B. kein erkennbares, leichtes oder hohes Verzerrungsrisiko) der einzelnen Studien zu bewerten (im Sinne von „Risk of bias“) und eine Qualitätsklassifizierung vorzunehmen.

Neben der Beurteilung des Verzerrungsrisikos auf der Ebene von einzelnen Studien ist außerdem eine Bewertung des gesamten Evidenzkörpers für die einzelnen Endpunkte entsprechend der GRADE-Methodik geplant. (23). Die GRADE-Methodik zur Bewertung der Qualität von Evidenz und Erstellung von evidenzbasierten Handlungsempfehlungen wurde von einer internationalen Arbeitsgruppe entwickelt ([www.gradeworkinggroup.org](http://www.gradeworkinggroup.org)) und wird mittlerweile von mehr als 70 Organisationen weltweit verwendet, u.a. der WHO und der Cochrane Collaboration.

## 5.4 Informationssynthese und –analyse

**Charakterisierung der Studien:** Diagnose- und Interventionsstudien werden nach Studientyp und Indikation (AMD und DR) getrennt dargestellt. Die Ergebnisse der Güte des OCT-Verfahrens (z.B. Sensitivität, Spezifität) werden aus den Publikationen extrahiert. Bei fehlenden Angaben wird, soweit möglich, eine eigene Berechnung der Werte erfolgen.

**Meta-Analyse:** Wenn es die Studienlage zulässt, ist eine quantitative Zusammenfassung der Einzelergebnisse in Form einer Meta-Analyse geplant (getrennt für Indikation, Fragestellung und Studientyp).

**Sensitivitätsanalyse:** Sensitivitätsanalysen sind insbesondere für Studien unterschiedlicher Qualität geplant.

**Subgruppenanalyse:** Subgruppenanalysen sind für Merkmale wie z.B. Type des OCT-Verfahrens (ältere vs neuere Modelle), Qualifikation / Erfahrung des Untersuchers, Ausprägung der AMD bzw. DR vorgesehen.

# Literatur

1. Berufsverband der Augenärzte Deutschlands. Statistische Datenbank: Augenkrankheiten. Zugänglich über http://www.augeninfo.de/stat_db/amd.php. Zugriff am 14. Dezember 2012.

2. Holz FG, Helb HM. Modern pharmacotherapy of age-related macular degeneration. Internist 2006;47:192-8.

3. Kahn HA, Leibowitz HM, Ganley JP, et al. The Framingham Eye Study. I. Outline and major prevalence findings. Am J Epidemiol 1977;106:17-32.

4. Vedula SS, Krzystolik M. Antiangiogenic therapy with anti-vascular endothelial growth factor modalities for neovascular age-related macular degeneration. Cochrane Database Syst Rev 2008;16:CD005139.

5. Ferris FL, 3rd, Fine SL, Hyman L. Age-related macular degeneration and blindness due to neovascular maculopathy. Arch Ophthalmol 1984;102:1640-2.

6. Schmucker C, Ehlken C, Hansen LL, et al. Intravitreal bevacizumab (Avastin) vs. ranibizumab (Lucentis) for the treatment of age-related macular degeneration: a systematic review. Curr Opin Ophthalmol 2010;21:218-26.

7. Schmucker C, Ehlken C, Agostini HT, et al. A safety review and meta-analyses of bevacizumab and ranibizumab: off-label versus goldstandard. PLoS One 2012;7:e42701. Epub 2012 Aug 3.

8. Heier JS, Brown DM, Chong V, et al. VIEW 1 and VIEW 2 Study Groups. [Intravitreal aflibercept (VEGF trap-eye) in wet age-related macular degeneration.](http://www.ncbi.nlm.nih.gov/pubmed/23084240) Ophthalmology 2012;119:2537-48.

9. Stellungnahme von DOG, Retinologischer Gesellschaft und BVA. Die Anti-VEGF-Therapie bei der neovaskulären altersabhängigen Makuladegeneration: Therapeutische Strategien Februar 2012. Zugänglich über http://www.dog.org/wp-content/uploads/2009/08/Stellungnahme-Anti-VEGF-Therapie-bei-der-neovaskul%C3%A4ren-Therapeutische-Strategie-Febr-2012-final.pdf. Zugriff am 14. Januar 2013.

10. Frank RN. Diabetic Retinopathy. N Engl J Med 2004;350: 48–58.

11. Nentwich MM, Ulbig MW. Diabetische Retinopathie. Der Diabetologe 2010;6:491.

12. Early Treatment Diabetic Retinopathy Study Research Group. Photocoagulation for diabetic macular edema: Early Treatment Diabetic Retinopathy Study report number 1: Arch Ophthalmol 1985;103:1796–1806.

13. Mitchell P, Bandello F, Schmidt-Erfurth U, et al. The RESTORE study: ranibizumab monotherapy or combined with laser versus laser monotherapy for diabetic macular edema. Ophthalmology 2011;118: 615–25.

14. Massin P, Bandello F, Garweg JG, et al. Safety and efficacy of ranibizumab in diabetic macular edema (RESOLVE Study): a 12-month, randomized, controlled, double-masked, multicenter phase II study. Diabetes Care 2010;33:2399–2405.

15. Kollias AN, Ulbig MW. Diabetische Retinopathie Frühzeitige Diagnostik und effiziente Therapie. Dtsch Arztebl Int 2010;107:75–84.

16. Stellungnahme der Deutschen Ophthalmologischen Gesellschaft, der Retinologischen Gesellschaft und des Berufsverbandes der Augenärzte Deutschlands zur Therapie der diabetischen Makulopathie Stand: Dezember 2010. Zugänglich über http://cms.augeninfo.de/fileadmin/stellungnahmen/17_01_2011_diabet_makulopathie.pdf. Zugriff am 14. Januar 2013.

17. Higgins JPT, Green S. Cochrane Handbook for Systematic Reviews of Interventions Version 5.1.0 [updated March 2011]. The Cochrane Collaboration 2011. Zugänglich über [www.cochrane-handbook.org](http://www.cochrane-handbook.org). Zugriff am 18. Januar 2013.

18. Rosenfeld PJ, Brown DM, Heier JS, et al. Ranibizumab for neovascular age-related macular degeneration. N Engl J Med 2006;355: 1419–1431.

19. Brown DM, Kaiser PK, Michels M, et al. Ranibizumab versus verteporfin for neovascular age-related macular degeneration. N Engl J Med 2006;355:1432-44.

20. CATT Research Group, Martin DF, Maguire MG, et al. Ranibizumab and bevacizumab for neovascular age-related macular degeneration. N Engl J Med 2011;364: 1897–1908.

21. IVAN Study Investigators, Chakravarthy U, Harding SP, et al. Ranibizumab versus bevacizumab to treat neovascular age-related macular degeneration: one-year findings from the IVAN randomized trial. Ophthalmology 2012;119:1399-411.

22. [Whiting PF](http://www.ncbi.nlm.nih.gov/pubmed?term=Whiting%20PF%5BAuthor%5D&cauthor=true&cauthor_uid=22007046), [Rutjes AW](http://www.ncbi.nlm.nih.gov/pubmed?term=Rutjes%20AW%5BAuthor%5D&cauthor=true&cauthor_uid=22007046), [Westwood ME](http://www.ncbi.nlm.nih.gov/pubmed?term=Westwood%20ME%5BAuthor%5D&cauthor=true&cauthor_uid=22007046), et al. QUADAS-2: a revised tool for the quality assessment of diagnostic accuracy studies. [Ann Intern Med](http://www.ncbi.nlm.nih.gov/pubmed/22007046) 2011;155:529-36.

23. Guyatt GH, Oxman AD, Vist GE, et al. GRADE: an emerging consensus on rating quality of evidence and strength of recommendations. BMJ 2008;336:924-6.
