# Supplementary material for: Treatment as Required versus Regular Monthly Treatment in the Management of Neovascular Age-Related Macular Degeneration: A Systematic Review and Meta-Analysis
Source: PLoS One. 2015 Sep 14;10(9):e0137866. doi: 10.1371/journal.pone.0137866 (PMC4569266; doi:10.1371/journal.pone.0137866)
Supplement: S1 Search Strategy — (DOCX) [file pone.0137866.s004.docx]

**Figure S1.** Systematic Literature Search in Medline (via OvidSP).

| **#** | **Searches** | **Results** |
| --- | --- | --- |
| 1 | exp Macular Degeneration/ | 16993 |
| 2 | exp Retinal Degeneration/ | 30968 |
| 3 | exp Retinal Neovascularization/ | 2318 |
| 4 | exp Choroidal Neovascularization/ | 4606 |
| 5 | exp Macula Lutea/ | 10127 |
| 6 | ((macul* or retina* or choroid*) adj5 degener*).tw. | 19715 |
| 7 | ((macul* or retina* or choroid*) adj5 neovasc*).tw. | 8371 |
| 8 | ((macul* or retina* or choroid*) adj5 neo-vasc*).tw. | 13 |
| 9 | amd.tw. | 6883 |
| 10 | cnv.tw. | 4951 |
| 11 | (subfoveal neovasc* or subfoveal neo-vasc*).tw. | 223 |
| 12 | (extrafoveal neovasc* or extrafoveal neo-vasc*).tw. | 9 |
| 13 | (juxtafoveal neovasc* or juxtafoveal neo-vasc*).tw. | 9 |
| 14 | (occult neovasc* or occult neo-vasc*).tw. | 34 |
| 15 | (classic neovasc* or classic neo-vasc*).tw. | 21 |
| 16 | (chor* neovasc* or chor* neo-vasc*).tw. | 5245 |
| 17 | or/1-16 | 53180 |
| 18 | Diabetic Retinopathy/ | 18919 |
| 19 | ((macula* adj3 edema*) or (macula* adj3 oedem*)).tw. | 6590 |
| 20 | retinopath*.tw. | 28700 |
| 21 | (CME or CSME or CMO or CSMO).tw. | 3989 |
| 22 | (DMO or DME).tw. | 1630 |
| 23 | 19 or 20 or 21 or 22 | 37837 |
| 24 | diabet*.tw. | 387114 |
| 25 | exp Diabetes Mellitus/ | 315240 |
| 26 | 24 or 25 | 439260 |
| 27 | 23 and 26 | 20195 |
| 28 | 18 or 27 | 25632 |
| 29 | 17 or 28 | 73535 |
| 30 | exp Vascular Endothelial Growth Factors/ | 40497 |
| 31 | Vascular Endothelial Growth Factor*.mp. | 54761 |
| 32 | vegf*.mp. | 44281 |
| 33 | (bevacizumab or avastin or ranibizumab or rhufab or lucentis or pegaptanib or macugen or eyelea or aflibercept).mp. | 13967 |
| 34 | or/30-33 | 66961 |
| 35 | 29 and 34 | 7133 |
| 36 | exp animals/ not humans/ | 4021923 |
| 37 | 35 not 36 | 6157 |
| 38 | (case reports or comment or editorial).pt. | 2401359 |
| 39 | 37 not 38 | 4821 |
| 40 | remove duplicates from 39 | 3426 |
| 41 | (eng or ger).la. | 18263141 |
| 42 | 40 and 41 | 3149 |
